# Endothelial derived miRNA-9 mediated cardiac fibrosis in diabetes and its regulation by ZFAS1

**DOI:** 10.1371/journal.pone.0276076

**Published:** 2022-10-14

**Authors:** Biao Feng, Jieting Liu, Eric Wang, Zhaoliang Su, Subrata Chakrabarti

**Affiliations:** 1 Department of Pathology and Laboratory Medicine, Western University, London, Ontario, Canada; 2 Lawson Health Research Institute, Ontario, Canada; 3 Mudanjiang Medical University, Mudanjiang, China; 4 Jiangsu University, Zhenjiang, China; Universita degli Studi della Campania Luigi Vanvitelli, ITALY

## Abstract

Diabetic cardiomyopathy (DCM) is one of the most prevalent causes of morbidity and mortality in diabetic patients. Hyperglycemia induces increased expression/deposition of extracellular matrix (ECM) proteins including fibronectin (FN) and collagen (Col) and plays an important role in fibrosis in diabetic cardiomyopathy (DCM). The roles of RNAs including microRNA (miRNA) and long non-coding RNAs (lncRNA) have begun to be understood in many conditions. In this study, we investigated the role of a specific miRNA, miR-9, and its interactions with lncRNA ZFAS1 in mediating fibrosis in DCM. Treatment with 25 mM glucose (HG) decreased miR-9 expression and increased expressions of ZFAS1, ECM proteins and inflammatory markers, compared to 5 mM glucose (NG) in the HCMECs by using qRT-PCR. Glucose-induced upregulation of ECM proteins can be prevented by ZFAS1 siRNA or miR-9 mimic transfection. Luciferase assay was confirmed miR-9 binding to FN 3’-UTR. miR-9 expression can be regulated by ZFAS1 through polycomb repressive complex 2 (PRC2) components using RNA immunoprecipitation (RIP) and chromatin immunoprecipitation (ChIP) assays. In the *in vivo* experiment, hyperglycemia-induced the ECM production can be prevented by the miR-9 overexpression in the fibrosis in DCM. These studies showed a novel glucose-induced molecular mechanism in which ZFAS1 participates in the transcriptional regulation of ECM protein production in diabetes through miR-9.

## 1. Introduction

Diabetes is a worldwide healthcare challenge. According to data from IDF, the number of adults affected with diabetes globally is estimated to reach 578 million by 2030. Patients with diabetes mellitus are at increased risk for developing to chronic complications, such as retinopathy, nephropathy and cardiomyopathy. Cardiomyopathy is one of the leading cause of morbidity and mortality among patients with diabetes [1, 2]. Diabetic cardiomyopathy (DCM) is characterized by left ventricular hypertrophy, myocardial fibrosis and diastolic dysfunction, which are not attributable to underlying coronary artery disease or hypertension [3]. One of the key manifestations of DCM, myocardial fibrosis, is caused by increased production and deposition of extracellular matrix (ECM) proteins, such as collagen and fibronectin (FN). Increased ECM protein deposition leads to structural changes in the heart in DCM include thickening of the capillary basement membrane, focal myocardial fibrosis etc. [4]. Endothelial cells (ECs) are initial targets of hyperglycemic damage and play a major role in the production of ECM proteins in all chronic diabetic complications [5]. Several regulatory mechanisms including specific microRNAs (miRNAs) and long non-coding RNAs (lncRNAs) may play significant roles increased ECM deposition in DCM.

miRNAs are short (~22nt) RNA molecules with no protein coding function, so-called non-coding RNAs. miRNAs regulate biological processes through interactions with the 3’UTR of target mRNAs. miRNAs have been demonstrated to contribute to pathological processes and the development of cardiac fibrosis in DCM [6]. We have previously shown downregulation of miR-146a in association with upregulation of ECM protein transcripts in the heart, kidneys and retinas in diabetes [7–9]. Other investigators also demonstrated the involvement of other miRNAs in the regulation of ECM protein expression in diabetes [10, 11].

Several studies showed that alteration of miR-9 expression related to the cancers, such as cervical cancer, breast cancer, prostate cancer, etc., and Alzheimer’s disease [12]. miR-9 also mediates CALHM1-activated ATP-P2X7R signalling in diabetic neuropathy in diabetic rat model [13]. One study showed that miR-9 inhibits high glucose-induced proliferation, differentiation and collagen accumulation of cardiac fibroblasts by down-regulation of TGFBR2 [14]. Previous studies suggest that methylation of miR-9-1 and miR-9-3 favors diabetic retinopathy (DR) in patients with diabetes [15]. However, role of miR-9 in DCM has not reported.

lncRNAs are RNA molecules longer than 200 nucleotides in length that are also not translated into functional proteins [16]. Apart from miRNAs, lncRNAs are another major class of non-coding RNAs that are widely expressed in mammals. lncRNAs regulate local and distal genes by various mechanisms and play key roles in diverse biological processes. They play important roles in physiological processes and cellular functions such as cell proliferation, resistance to apoptosis, and induction of angiogenesis [17, 18]. Recent evidence shows that dysregulation of target genes leads to abnormal lncRNA expression in diabetes [19]. A previous study from our lab showed that changes in expression of the lncRNA ANRIL plays an important role in DCM [20]. Nevertheless, the potential functions of lncRNAs and the related mechanisms in DCM are still largely unknown and needs further exploration.

Recent evidences have highlighted the importance of epigenetic regulation in the pathogenesis of DCM [21–23]. The research data further indicate that certain epigenetic processes are fine-tuned by lncRNAs via direct interaction with cis-regulatory elements, or through interactions with key RNA binding proteins and chromatin modifiers [24]. Such epigenetic modifications include DNA methylation, acetylation and methylation of histones. These modifications can alter the production of specific miRNAs and lncRNAs, which play important roles through regulation of gene expression at both transcriptional and translational levels [25]. Furthermore, a regulatory interaction exists between specific miRNA and lncRNA ANRIL [26].

In this study, we examined the role of miR9 in diabetic conditions both *in vitro* and *in vivo*. To examine the role of miR-9 in DCM in vitro, we used endothelial-specific miR-9 overexpressing mice generated by us. We also investigated the role of ZFAS1 in regulating the production of ECM protein in human cardiac microvascular endothelial cells (HCMECs) and in DCM in mouse models. We further examined the interactions between ZFAS1 and miR-9 in mediating cardiac fibrosis in diabetes.

## 2. Materials and methods

### 2.1 Cell culture

Human cardiac microvascular endothelial cells (HCMECs) were obtained from ScienceCell (Carlsbad, CA, USA) and were cultured in endothelial basal media-2 (EBM-2) supplemented with Endothelial Cell Growth Medium-2 SingleQuots (EGM-2) and 10% fetal bovine serum as previously described [7, 27]. Cells were serum-starved for 24 hours once they reached 80–90% confluency. HCMECs were then treated with serum-free media containing various concentrations of D-glucose (with 25 mM mannitol as osmotic control) for 48 hours. The cells were then collected for the further analysis.

Mouse heart endothelial cells (MHECs), fibroblasts and myocytes were isolated as described previously [7, 27]. Briefly, on the day of the experiment, Dynabeads were washed in 0.1% BSA-PBS (B-PBS) for 3 times. The beads were then incubated with 20 μL of rat anti-mouse CD31 monoclonal antibody (Invitrogen, ON, Canada) at 4°C for 3 hours. Anti-CD31 antibody-conjugated Dynabeads (CD31-beads) were washed for 3 times using B-PBS and re-suspended in 200 μL B-PBS on ice for later use. The mice were euthanized, the heart tissues were collected, and tissue from the left ventricles were minced into 1 mm^2^ small pieces and digested with collagenase A for one hour at 37°C. The mixtures were flushed using 5 ml pipet and transferred to 50 mL tube containing cold DMEM with 2% FCS (DMEM-F) and pelleted by centrifugation. The pellets were re-suspended in the DMEM-F solution, passed through a 100 μm cell strainer, and pelleted again. The cells were further incubated with CD31-beads for one hour at 4°C and washed 5 times using B-PBS. Cells bound to CD31-beads (MHECs) were cultured in 2% gelatin coated 6 well plates (Falcon, BD, Canada) in EBM2 with supplemental kit same as above (Lonza, MD, USA). The unbound cells were incubated in 75cm^2^ flask at 37°C for one hour, after which the suspended cells (myocytes) were collected. Cells attached to the bottom of the flask (fibroblasts) were cultured with DMEM with 10% FCS and collected after purification using CD90 beads. Isolated cardiac ECs (estimated purity 90%), fibroblasts and myocytes were used for separate analyses. The cellular phenotypes were also confirmed by RT-PCR assay for cell type specific markers.

### 2.2 EC cell experiments

HCMECs were transfected with miRNA-9 mimic (20 nM) using Lipofectamine 3000 (Invitrogen Canada, ON, Canada). Scrambled mimics was used as a control. miRNA transfection efficiency was determined by TaqMan real-time RT-PCR technique [7, 28]. For siRNA experiment, ECs were transfected with ZFAS1 siRNA (20 nM) using Lipofectamine 3000. We initially used multiple siRNAs. They produced similar results and then we selected one with best results. Scrambled siRNA was used a control. For methylation inhibition experiments, 3-Deazaneplanocin A (DZNep, 5 μM, Cayman Chemical, Ann Arbor, MI, USA) was applied as described in previous studies to HCMECs for 1 hour prior to the addition of D-glucose [7, 27, 29]. Treated HCMECs and their controls were then subjected to various glucose treatments, then collected after 48 hours of glucose incubation. Cell viability was examined using MTT assay.

### 2.3 Animals

All animal experiments were performed in accordance with the Canadian Council on Animal Care. The protocols were approved by Western University Animal Care and Veterinary services. This investigation conforms to the Guide for the Care and Use of Laboratory Animals published by the US National Institutes of Health (NIH Publication No. 85–23, revised 1996).

### 2.4 Generation of miR-9 transgenic mice

We generated miR-9 transgenic (miR9TG) mice with EC-specific promoter using previously described methodology [27]. A cDNA fragment containing miR-9 was inserted into the pg52pSPTg.T2FpAXK (pg52) plasmid which contains a Tie2 promoter, enhancer and an SV40 PolyA signal (kindly provided by Dr Sato, Nara Institute of Science and Technology Graduate School of Biological Sciences, Ikoma, Japan) [4]. The fragment containing Tie2-miR-9 was excised by using restriction enzyme SalI, purified, and then injected into mouse blastocysts from the C57BL/6 background. These blastocysts were then transferred into pseudopregnant female mice [28]. Transgenic mice founders were identified by PCR-based assays using the genomic DNA from tail-tip biopsy specimens according to the protocol described previously [27]. miR9TG mice for the experiments were also confirmed by the same method (S1 Fig). No behavioural or phenotypical alterations were observed in the transgenic mice.

We used miR9TG mice and littermate controls (both of C57BL/6 background) mice (8 weeks) in this study. Transgenic and control mice were further randomly divided into diabetic and control groups. The animals were injected with freshly prepared streptozotocin (STZ) in sodium citrate buffer (I.P., pH 4.5, 50 mg/kg) or an equal volume of buffer only once a day for 5 consecutive days. Diabetes was confirmed by measuring blood glucose levels (>16.7 mmol/L) following the final STZ injection as described previously [28]. Following confirmation of diabetic for 2 months, the mice were sacrificed using isoflurane and cardiac tissues were collected. A small portion of ventricular tissue was fixed in 10% neutral-buffered formalin and were embedded in paraffin. After fixing 48 hours, the tissues were cut in 5 μm sections and stained with hematoxylin-eosin and trichrome for histologic analysis. The remainder of the cardiac tissues were stored at -80°C for later use.

### 2.5 miRNA analysis

miRNAs were extracted from the cells and tissues using the mirVana miRNA isolation kit (Thermo Fisher Scientific, MA, USA) following manufacturers’ instructions. Briefly, the cells were lysed and the tissues were homogenized using the Lysis/Binding solution. The miRNA additive from the kit was added to the lysed samples. Equal volume acid-phenol:chloroform was added to cell suspension solution. Following centrifugation, the aqueous phase was removed and 1.25-fold 100% ethanol was added to the mixture. The mixture was then passed through the filter column and miRNAs were eluted. Reverse transcription of RNA and real-time PCR (RT-PCR) were performed using a kit (Life Technologies, USA) as follows: 10 μL TaqMan 2X Universal PCR Master Mix, 8 μL Nuclease-free water, 1 μL TaqMan microRNA assay (Life Technologies, CA, USA) and 1 μL RT-product. U6 snRNA was used as an internal control [27].

### 2.6 mRNA analysis

Total RNA was extracted using TRIzol^™^ reagent (Invitrogen Canada Inc., ON, Canada) as previously described [27, 28]. Total RNA (1 μg) was used for cDNA synthesis by using high capacity cDNA reverse transcription kit (ThermoFisher, CA, USA). Real-time quantitative RT-PCR was performed using the LightCycler (Roche Diagnostics Canada, QC, Canada) and normalized to β-actin mRNA to control the amount of template in the reaction mixtures. The primer sequences are shown in Table 1.

**Table 1:** The primers for the experiments.

Gene	Primer sequence (5’-3’)	Product size
Hsa/Mmu β-actin	TGTGGATCAGCAAGCAGGAG TGCGCAAGTTAGGTTTTGTC	120bp
Hsa FN1	GATAAATCAACAGTGGGAGC CCCAGATCATGGAGTCTTTA	184bp
Hsa Col 1a1	GAGGGCCAAGACGAAGACATC CAGATCACGTCATCGCACAAC	140bp
Hsa Col 4a1	CAAGAGGATTTCCAGGTCCA TCATTGCCTTGCACGTAGAG	187bp
Hsa NF-kB	TGGAGCAGGCTATCAGTCA GCACAGCATTCAGGTCGTA	96bp
Hsa IL6	GGGGCTGCTCCTGGTGTTG CTGAGATGCCGTCGAGGATGTA	149bp
Hsa IL-1 β	GCGGCATCCAGCTACGAATCT GGGCAGGGAACCAGCATCTT	109bp
Hsa EZH2	CCACCATTAATGTGCTGGAA TTCCTTGGAGGAGTATCCACA	369bp
Hsa EED	GCAACTGTAGGAAGCAACAGA CATAGGTCCATGCACAAGTGT	124bp
Hsa SUZ12	TACGGCTCCTATTGCCAAAC TGCTTCAGTTTGTTGCCTTG	226bp
Mmu FN1	CGGTAGGACCTTCTATTCCT GATACATGACCCCTTCATTG	335bp
H Mmu Col 1a1	CACCCTCAAGAGCCTGAGTC GTTCGGGCTGATGTACCAGT	253bp
Mmu Col 4a1	ACAGGCACAAGTTAAGGAAA ATCTCCTTTCTCTCCCAAAG	304bp
The primers for human FN 3’UTR mutation		
FN1Hsa(+)	AGAGCTC ATCATCTTTCCAATCCAGAGGAAC	
FN1Hsa mut(3)	AGGATATC TCCTCCAGAGCAAAGGGCTTAAG	
FN1Hsa mut(5)	AGGATATC CCAGCTTCAGCTCAACTCACAGC	
FN1Hsa(-)	TCTAGAAAGCTT TAATCACCCACCATAATTATACC	

Note: Hsa = human, Mmu = mouse

### 2.7 Protein extraction and ELISA

The cells were lysed in RIPA buffer (MilliporeSigma, Canada). Total protein was collected and the concentration was measured by using BCA kit (Thermo Fisher Scientific Inc., IL, USA). ELISAs for human FN and IL6 were performed using a commercially available kits (R&D Systems and Millipore Corporation, USA.) according to the manufacturer’s instructions.

### 2.8 Luciferase reporter assay

For luciferase reporter assay, a human FN 3’-UTR segment was amplified by PCR and inserted into the pMIR REPORT Luciferase vector with CMV promoter (Life Technologies, CA, USA) by using the Sac I and Hind III sites immediately downstream from the stop codon of luciferase. The following sets of primers were used to generate specific fragments for human FN 3’-UTR, forward primer, 5’-AGAGCTC TCATCTTTCCAATCCAGAGGAAC-3’; reverse primer, 5’-TCAAGCTT TAATCACCCACCATAATTATACC-3’ (underlined sequences indicate the endonuclease restriction site). Nucleotide substitutions were introduced by PCR to yield a mutated binding site. The primer sequences for human FN 3’UTR mutation cloning are listed in Table 1. The sequence of the cloned product was confirmed by sequencing analysis. The pMIR-FN 3’UTR, miR-9 mimic, and β-galactosidase control plasmids were then co-transfected into HEK293A cells for 24 hours. After transfection, luciferase activity was measured using the Dual-Light Chemiluminescent Reporter Gene Assay System (Life Technologies, CA, USA) and Chemiluminescent SpectraMax M5 (Molecular Devices, Sunnyvale, CA) following the manufacturer’s instructions [8, 30]. Luciferase activity was normalized for transfection efficiency by measuring β-galactosidase activity. The experiments were performed in triplicates.

### 2.9 RNA immunoprecipitation (RIP)

Cell lysates from HCMECs cultured in NG or HG were used for immunoprecipitation using the Magna RIP RNA-binding protein immunoprecipitation kit (Millipore, ON, Canada) following the manufacturer’s instructions. Anti-IgG (control) and anti-EZH2 antibodies (Millipore, Canada) were used to co-precipitate the RNA-binding proteins of interest. The extracted RNAs were then reverse transcribed to cDNA, and analyzed by RT-PCR.

### 2.10 Chromatin immunoprecipitation (ChIP)

ChIP assays (Milipore, CA, USA) were carried out as previously described [29]. Briefly, HCMECs cultured in NG or HG were collected for immunoprecipitation. Cells were then fixed with 1% formaldehyde and then lysed. ChIP assays were performed using anti-IgG and anti-EZH2 (Millipore, Canada) antibodies. Anti-mouse IgG was used as a negative control. The immunoprecipitated DNA was detected by RT-qPCR using promoter-specific primers for miR-9 promoter region: forward: 5’-GAAATGGGACTGTGACTCCTAC-3’, reverse: 5’-AGAGGATACAAGAGGAGGAGAG-3’ [31].

### 2.11 Histology staining

The mouse heart tissues were collected, fixed and embedded in paraffin and cut it to 5 μm thickness section on positively charged slides. The sections were deparaffinized in xylene and stained with hematoxylin and eosin, or with Masson’s trichrome stain for evaluation as described previously [27, 32].

### 2.12 Statistical analysis

Data were expressed as mean ± standard error (SEM) and statistical significance of results were analyzed by ANOVA and Student’s t-test as appropriate. A p value of 0.05 or less (p < 0.05) was considered significant between 2 groups. The results were expressed as average of n = 6–8 animals per group.

## 3. Results

### 3.1 miR-9 regulates glucose-induced increase in ECM protein expression in endothelial cells

**Fig 1:**
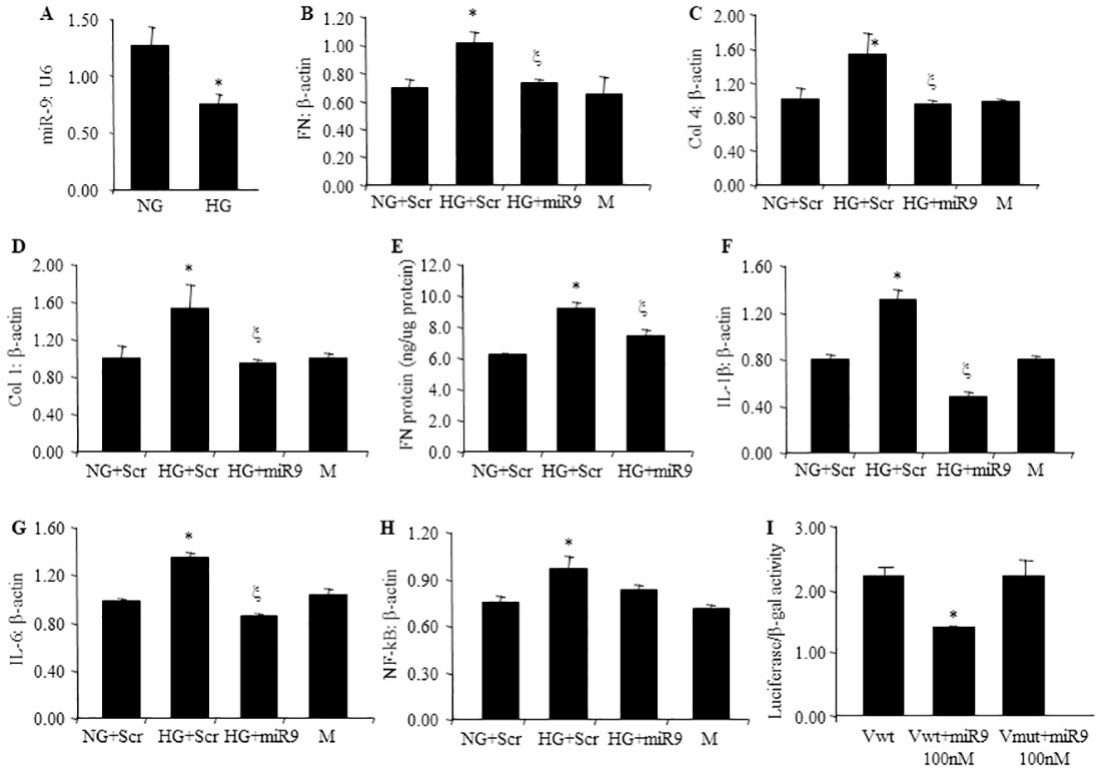
miR-9 regulates glucose-induced production of ECM in vitro in HCMEC. RT-qPCR analyses indicating HG-induced downregulation of (A) miR-9 transcript, (B, C, D) ECM transcripts (FN, Col4a1, Col1a1) and (E) FN protein expression in HCMECs. Similar glucose induced effects were also seen regarding proinflammatory markers (F), IL-1 β, (G) IL-6, and H) NF-kB1. Such glucose-induced increases of these ECM were prevented following (B-H) miR-9 mimic transfection Luciferase reporter assay demonstrated the interaction between miR-9 and FN(I). (mRNA expressed as a ratio to β-actin; *p < 0.05, compared to Scr HG; Scr = scrambled mimic; NG = 5 mM D-glucose; HG = 25 mM D-glucose; M = 25 mM mannitol; Vwt = wild pMIR vector; Vmut = mutant pMIR vector; n = 6).

As glucose induced alterations of endothelial cells is the key initiating factor for tissue damage in diabetes [33], we examined HCMECs for glucose induced changes. Initially we confirmed our previous finding that glucose-induced FN overexpression peaks at 48 hrs (not shown). Hence, we used this time point for all subsequent experiments. We first studied the expression of miR-9 in HCMECs after exposure to HG. miR-9 expression was decreased after treatment with HG for 48 hours (Fig 1A). In parallel, expressions of ECM proteins’ mRNA (FN, collagen 4α1, Collgen1α1,) were increased after exposure to HG at the same time point (Fig 1B–1D). Changes in mRNA expressions were mirrored by protein expression levels (Fig 1E).

To establish a cause–effect relationship we examined the effect of miR-9 overexpression. miR-9 mimics transfection prevented glucose-induced upregulation of ECM protein production on both the mRNA and protein levels (Fig 1B–1E). Similarly, glucose induced augmented expression of inflammatory mediators (IL-1β, IL6) and transcription factor (NF-κB) were also prevented by miR-9 mimic transfection (Fig 1F–1H). To examine a direct regulatory relationship of miR-9 on its target gene, FN 3’UTR fragment was cloned to pMIR vector, after co-transfection with miR-9 mimics in to HEK293 cells for 24 hours, luciferase reporter activity was found to be significantly decreased in the wild type FN 3’UTR transfection group compared to the mutant FN 3’UTR transfection group (Fig 1I). These findings further supported a direct interaction between miR-9 and FN 3’UTR. No alteration in cell viability were seen in MTT assay (not shown).

### 3.2 Effects of miR-9 on glucose induced ECM protein and pro-inflammatory factor expressions are regulated by ZFAS1

**Fig 2:**
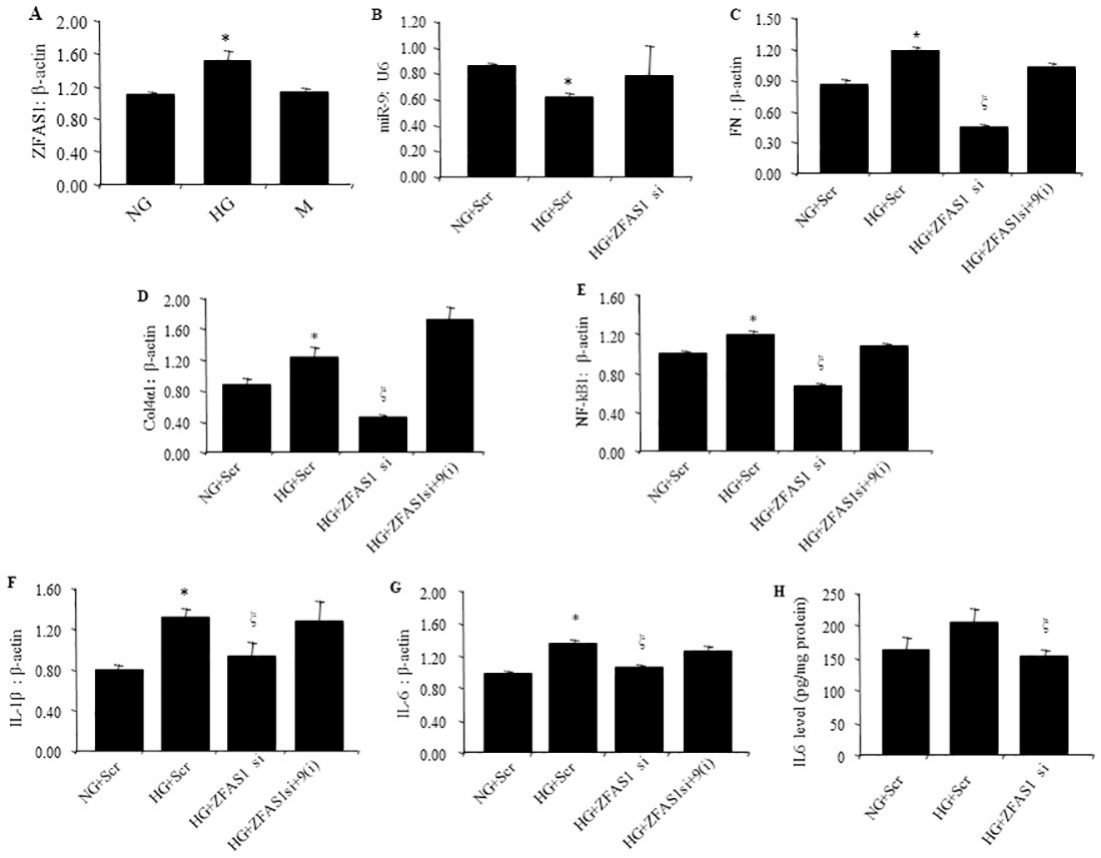
Effects of ZFAS1 silencing on mRNA levels of ECM and proinflammatory factors in HCMECs. (A) ZFAS expression was increased in HG along with B) reduced miR9 expression., Treatment of HCMECs with HG also increased the mRNA expression of ECM proteins C) FN and D) Col 4a1, proinflammatory factors markers E) NF-kB1 (F), IL-1 β and (G) IL-6, compared with NG along with H) IL-6 protein levels. Suppression of ZFAS1 reversed these alterations (B-H). Rescue experiments showed that (C-G) ZFAS1siRNA induced recovery can be reversed by miR-9 antagomir (A-E). (RNA data are expressed as a ratio to β-actin; mean ± SEM. *p<0.05 vs NG+Scr; ξ p<0.05 vs HG+Scr. NG = 5 mM D-glucose; HG = 25 mM D-glucose; M = 25 mM mannitol; Scr = scrambled siRNA; si = siRNA; 9(i) = miR-9 antagomir; n = 6).

As previously mentioned, the lncRNA ZFAS1 may regulate miRNA action. Hence, we examined whether nullifying the effects of ZFAS1 has any effect on miR-9 and the downstream targets. We first confirmed that high glucose upregulates ZFAS1 RNA expression (Fig 2A). We found that glucose-induced mRNA overexpression of ECM proteins including FN, collagen 4a1 was prevented by ZFAS1 silencing via siRNA transfection (Fig 2C and 2D), in association with correction of glucose induced reduced miR-9 levels (Fig 2B). We further measured the expressions of the pro-inflammatory factors (NF-κB, IL-6 and IL-1β). The expressions of these pro-inflammatory factors were also corrected following ZFAS1 silencing (Fig 2E–2G). Such changes were further reflected in IL-6 protein levels (Fig 2H).

We further explored whether ZFAS1 acted through interactions with miR-9 by carrying out a rescue experiment. For this experiment, we transfected HCMECs using ZFAS1 siRNA, then transfected using miR-9 antagomir. We found that such approach rescued ZFAS1 siRNA-mediated suppression of the aforementioned factors at mRNA and protein levels (Fig 2C–2G). These data indicate that lncRNA ZFAS1 mediates its action through miR-9 in the current scenario.

### 3.3 Polycomb repressive complex 2 (PRC2) plays a role in ZFAS1-miR-9 pathway

To further understand the mechanistic actions of ZFAS1 and miR-9, we used *in vitro* experiments. It has been shown that ZFAS1 and polycomb repressive complex 2 (PRC2, a histone methyltransferase) have a regulatory relationship in other systems [34]. Hence, we examined such relationship in this context. We used a global histone methylation inhibitor, 3-deazaneplanocin A (DZNep). PRC2 has methyltransferase activity and is one of two classes of polycomb-group proteins. PRC2 has three subunits: EZH2, EED and SUZ12 [35, 36].

**Fig 3:**
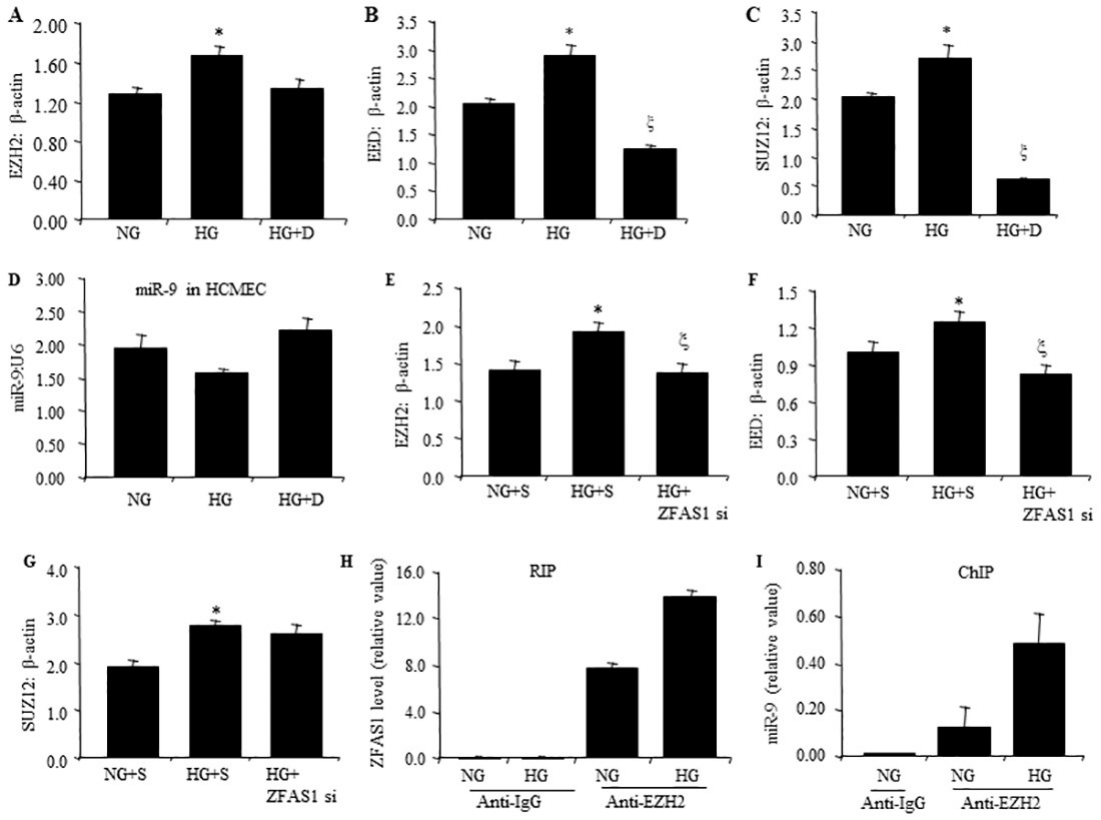
ZFAS1–PRC2 complex interaction regulating miR-9 expression in vitro in HCMEC. Treatment of HCMECs with methylation inhibitors (DZNep) reduced HG induced mRNA upregulation of (A, B, C) EZH2, EED and SUZ12. In conjunction, DZNep also corrected HG induced decreased (D) miR-9 levels. Interestingly, transfection of HCMECs with ZFAS1 siRNA reduced HG induced (E, F) upregulation of EZH2 and EED mRNA, whereas(G) SUZ12 mRNA remained unchanged. (H) RIP assay using anti-EZH2 antibody performed in HCMECs showed glucose-induced increased binding of ZFAS1 with EZH2 (anti-IgG as negative control). (I) ChIP assay demonstrated increased binding of EZH2 on miR-9 promoter as measured by RT-PCR. (NG = 5 mM glucose; HG = 25 mM glucose; RIP = RNA immunoprecipitation; ChIP = chromatin immunoprecipitation RNA; D = DZNep; data expressed as a ratio to β-actin, mean ± SEM; *P < 0.05 compared to NG; ξ < 0.05 compared to HG; n = 6/group).

DZNep corrected glucose-mediated augmentation of two PRC2 components namely, EZH2, EED and SUZ12 mRNAs (Fig 3A–3C) and recovered glucose induced miR-9 downregulation (Fig 3D). We then tested the effects of ZFAS1 on polycomb repressive complex 2 (PRC2), by using ZFAS1 siRNA to silence ZFAS1. Silencing of ZFAS1 led to the repression of enhancer of zeste homolog 2 (EZH2) and EED, but not SUZ12 (Fig 3E–3G).

Subsequently, we performed RIP and ChIP analyses using anti-EZH2 antibodies and found that ZFAS1 binding to EZH2 (PRC2) was enhanced under high glucose conditions (Fig 3H). Furthermore ChIP analysis showed enhance interaction of EZH2 (PRC2 complex)-ZFAS1 with miR-9 promoter in high glucose (Fig 3I). Collectively these analyses indicate that ZFAS1 regulates miR-9 through PRC2 complex.

### 3.4 miR-9 prevents diabetes induced fibrosis in mouse heart

**Fig 4:**
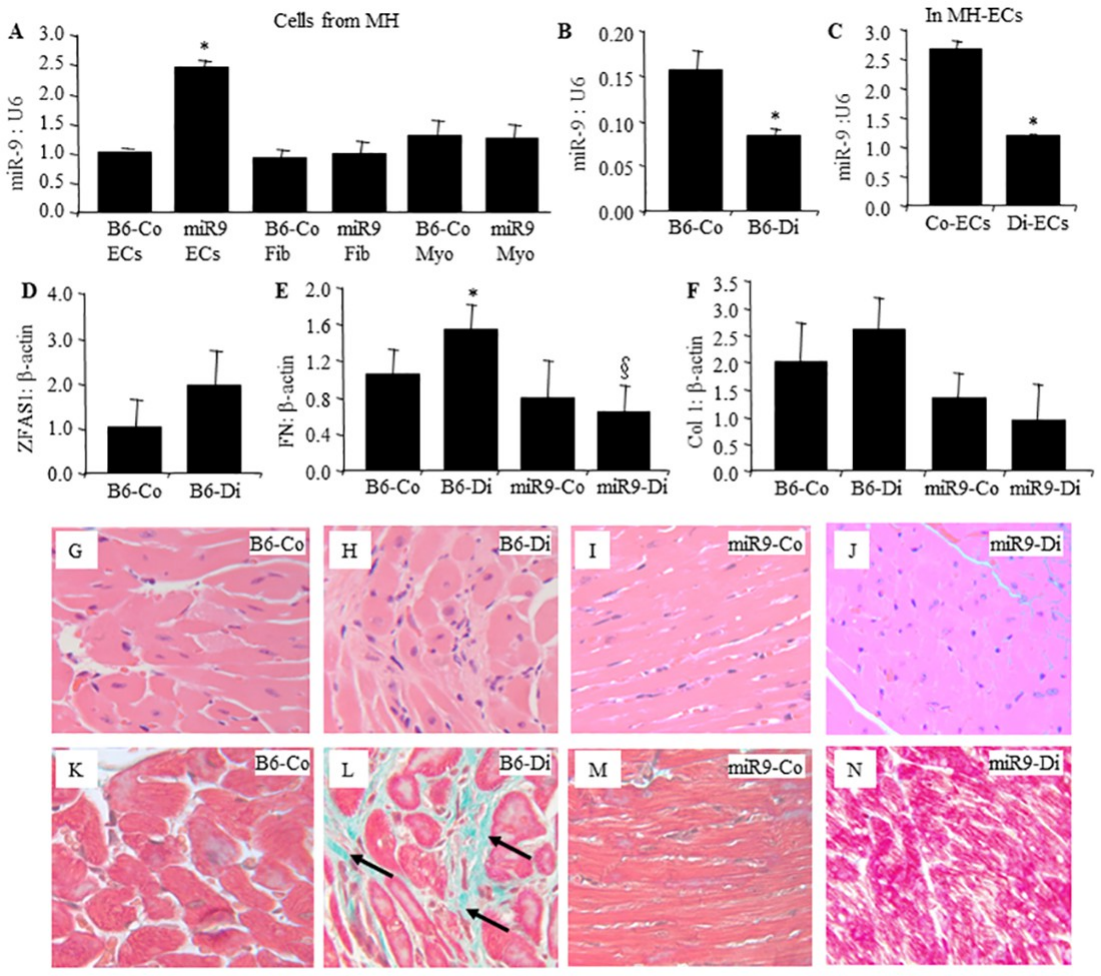
EC-specific miR-9 transgene (TG) alleviates diabetes-induced ECM (FN, Col1a1) production in vivo. A) RT-qPCR analyses of the cells from the cardiac tissues of the transgenic mice confirmed 2.5-fold increased miR-9 expression in the ECs but not in other cell types. Following two months of diabetes, reduced miR-9 were seen in the B) whole heart and in the C) ECs isolated from wild type diabetic mouse heart compared to the wild type non-diabetic controls, in association with increased expressions of (D) ZFAS1, ECM transcripts E) FN and F) Col1a1. Such diabetes induced ECM changes were prevented (E, F) in the miR-9 TG mice with diabetes. (G-J) hematoxylin and eosin and (K-N) corresponding trichrome staining showed increased focal fibrosis (arrow, green stain in L) in the hearts of wild diabetic animals compared with K) wild type control animals. Such diabetes-induced changes were not seen in the M.N) TG mice with or without diabetes. (Data expressed as a ratio to β-actin normalised to control; *P < 0.05, compared to B6-Co or Co-ECs; §P < 0.05, compared to B6-Di; B6-Co = Wild type non-diabetic controls; B6-Di = Wild type diabetic; miR9-Co = miR-9 transgenic control; and miR9-Di = miR-9 transgenic diabetic. MH = mouse heart. Myo = myocytes; fib = fibroblast; EC = endothelial cells. Co = control; Di = diabetic; n = 8/group. Same magnification for all micrographs).

We then explore whether such miR-9 mediated mechanisms play a role in a clinically relevant model of DCM. Fibrosis is one of the characteristics in DCM. To examine the potential role of miR-9 in this process, we generated miR-9 endothelial specific transgenic mice. We isolated various cells from mouse hearts, and confirmed miR-9 overexpression in the cardiac endothelial cells, but not in the fibroblasts and myocytes (Fig 4A).

Diabetic animals showed hyperglycemia, glycosuria compared to control animals, these parameters were not affected by miR-9 overexpression. Clinical data have been depicted in Table 2. We then examined expressions of miR-9 and ZFAS1 in the mouse hearts and ECs from mouse heart (Fig 4B–4D). We found that the expression of miR-9 was downregulated and ZFAS1 was upregulated in the hearts of diabetic mice compared to control mice. ECM protein expressions (FN and Col1) were also examined in cardiac tissues. mRNA expressions of both FN and Col1 were increased in the hearts of wild-type mice with diabetes (Fig 4E and 4F). All such abnormalities were prevented in the miR-9 TG mice with diabetes (Fig 4E and 4F). In parallel, focal myocardial fibrosis was present in the hearts of wild-type diabetic animals (Fig 4H) and were prevented in the transgenic mice with diabetes (Fig 4J).

**Table 2:** Parameters for clinical monitoring.

	Body Weight (g)	Blood Glucose (mmol/L)
C57BL/6-Control	30.3±3.3	7.3±0.6
C57BL/6-Diabetic	20.4±2.1*	30.2±3.6*
miR9 TG-Control	25.8±2.5	8.0±1.2
miR9 TG -Diabetic	22.0±1.5†	27.3±4.2†

Note: n = 6 to 8

[* *p* < 0.05 compared to C57BL/6 control group,

^†^ *p* < 0.05 compared to miR9 TG-control group]

## 4. Discussion

Our study demonstrated that endothelial derived miR-9 plays a key role in the pathogenesis of DCM. We have demonstrated that it regulates glucose induced increased ECM protein and inflammatory molecule production in the ECs. Using an EC specific miR-9 TG model, we further showed that such EC derived miR-9 regulate cardiac fibrosis in diabetes. In addition, we demonstrated a novel pathway in which lncRNA ZFAS1 regulates miR-9, to produce effects of glucose through PRC2 complex.

Hyperglycemia-induced ECM overproduction could alter the structure and function of the heart in diabetes [37]. Studies have shown that several miRs play important roles in endothelial to mesenchymal transition (EndMT) in DCM [38]. Our research has previously demonstrated that miR-200b regulates diabetes-induced EndMT in the heart and retina [28]. We have also shown that miR-146a influences cardiac fibrosis in diabetes [7]. Others have shown a protective role of miR-30c in cardiac metabolism in diabetes [39]. We have also shown that other miRs, including miR-1 and miR-133 are protective of cardiac hypertrophy in diabetes [40, 41]. Our present and previous studies have demonstrated that regulation of miRs in endothelial cells, the primary target of glucose-induced vascular damage in diabetes, can prevent cardiac structural and functional changes through the anti-inflammatory mechanism [7, 27]. These findings indicate that endothelial cell dysfunction is probably a primary target of glucose induced cardiac damage in diabetes.

MiRs possess a wide range of functions and are involved in most, if not all, chronic disease processes. MiR-9 is evolutionary conserved and play roles in various cellular activities in a context-dependent manner [42]. miR-9 is known to target important ECM proteins (fibronectin [FN], collagen [COL]) and their regulator (TGFβ1), multiple inflammatory mediators (IL-6, IL-1β, TNFα) and NF-*κ*B—key molecules in DCM [43]. Hence, miR-9 may act as a common mediator that regulates cardiac inflammation and subsequent fibrosis in DC. Preliminary studies have demonstrated downregulation of miR-9, along with upregulation of inflammatory mediators, transcription factors and ECM proteins in ECs incubated with HG and in the hearts of diabetic animals (Fig 1).

Another interesting part of the study is the identification of a novel regulatory mechanism. In this study, we have further confirmed that the lncRNA ZFAS1 regulates miR-9 through the PRC2. This is in keeping with similar regulation of other miRs. It is however interesting to note that silencing of ZFAS1 led to prevention of glucose induced upregulation of EZH2 and EED, but not SUZ12. Exact reasons for such findings are not clear. Further investigations may be needed to understand such phenomenon further.

LncRNAs regulate gene expression at the epigenetic, transcriptional and translational levels in a variety of ways. Histone methylation and acetylation are implicated good epigenetic marks in diabetic complications [44, 45]. Histone methylation is a process by which methyl groups are transferred to amino acid residues of histone proteins. EZH2, the catalytic core subunit of PRC2, acts as an epigenetic silencer through the trimethylation of lysine 27 on histone H3. Moreover, EZH2 is implicated in promoting tumour angiogenesis [46, 47]. DZNep was reported to selectively inhibit trimethylation of lysine 27 on histone H3 (H3K27me3) and lysine 20 on histone H4 (H4K20me3) as well as reactivate silenced genes in cancer cells [48]. In our experiments, increased levels of ECM protein were accompanied by EZH2 upregulation in HCMECs. Histone methylation blockade resulted in reduction in ECM mRNA expression, showing histone methylation is associated with HG induced increased ECM.

Although there are no previous reports on ZFAS1 alterations in diabetic cardiomyopathy, recent studies have shown that other lncRNAs play an important role in cardiac fibrosis. One study indicated that H19 negatively modulated the expression of DUSP5 gene in cardiac fibroblasts (CFs) and fibrotic tissues [49]. The study showed that the lncRNA myocardial infarction associated transcript (MIAT) is involved in myocardial infarction (MI). When MIAT is upregulated, it is accompanied by miR-24 down-regulation and TGF-β1 up-regulation [50]. The expression of MIAT was also significantly upregulated in mouse CFs treated with Ang II [50]. Our previous study showed that ANRIL knock out can prevented the elevated expressions of extracellular matrix (ECM) products in the tissues of heart and kidney in diabetic animals, and we also demonstrated the interaction of ANRIL with EZH2 (PRC2) complex in the regulation of VEGF [26]. MALAT1 also played an important role in the pathogenesis of chronic diabetic complications involving the heart and kidneys [51].

LncRNA ZFAS1 is highly expressed in the heart and is a regulator of organ development, cancer growth and metastasis, apoptosis and cell cycle regulation [52, 53]. ZFAS1 was originally discovered to play a vital role in hepatocellular carcinoma progression; there ZFAS1 may be a potential tumor suppressor [54]. ZFAS1 is located in the 20q13.13 region, which functions in breast cancer progression [55]. LncRNA ZFAS1 has also been reported to regulate cell proliferation, migration and invasion in bladder cancer by targeting miR-193a-3p/SDC1 [56]. However, the biological function and molecular mechanisms of ZFAS1 in diabetes remained unclear. Therefore, understanding the effects of ZFAS1 on diabetes can increase the essential knowledge and provide novel way to the diagnostic and treatment of diabetic patients. In our study, we found that ZFAS1 regulates miR-9 by binding to the PRC2 complex, subsequently regulating expressions of ECM and proinflammatory factors in DCM. However exact binding site of the PRC complex has not been characterised and need further investigation.

**Fig 5:**
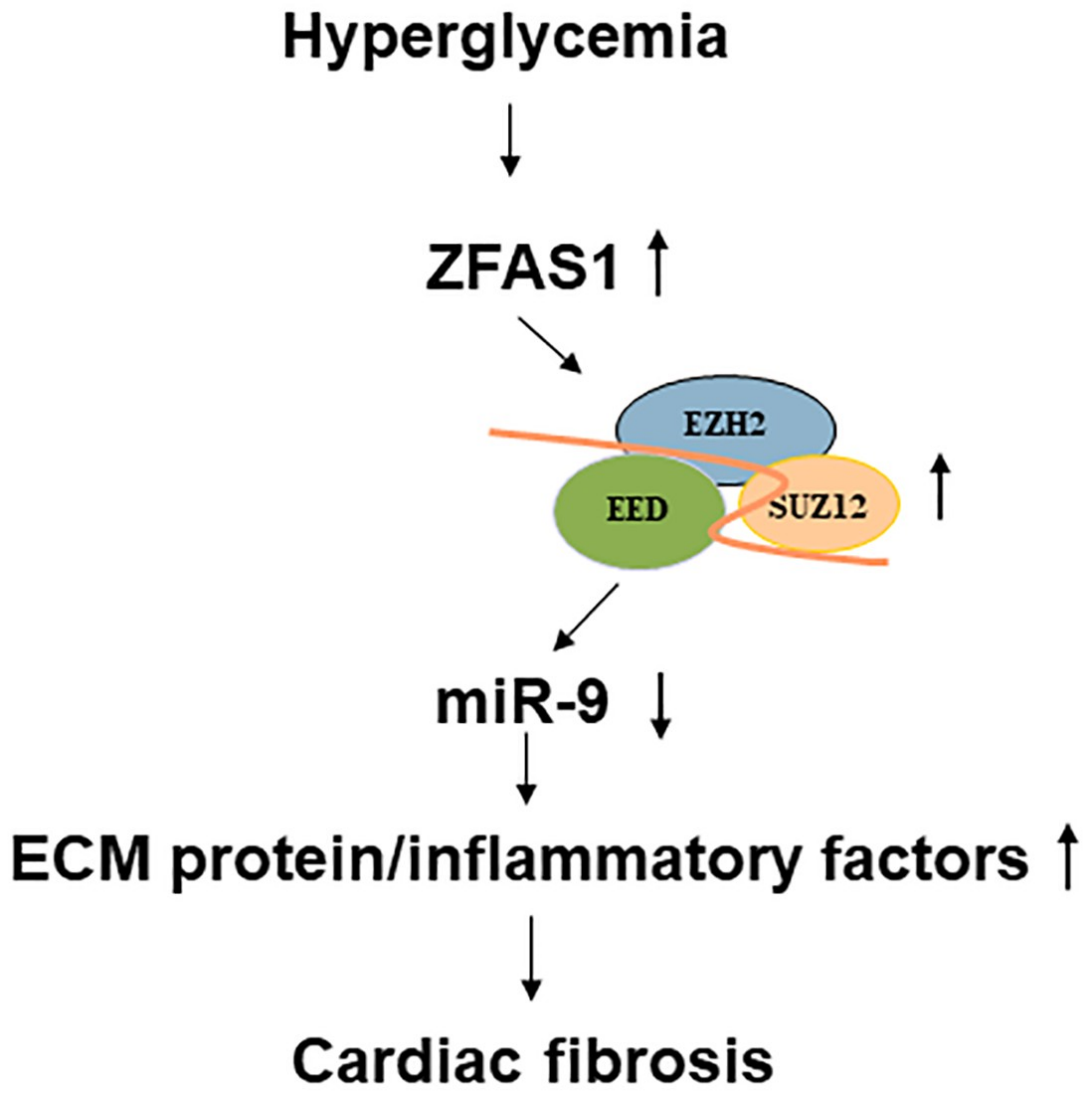
A schematic outline of the mechanism related to ZFAS1 in cardiac fibrosis in DCM.

In summary, we showed that glucose causes upregulation of ZFAS1 in the human cardiac microvascular endothelial cells and in the heart in diabetic animals. This upregulation is responsible for altered the expressions of ECM protein production and proinflammatory factors. ZFAS1 promoted ECM protein production by regulating PRC2 components and inhibiting miR-9 expression. Data from this study shed light on a potentially novel method to prevent DCM using an RNA based approach. A schematic of the regulatory process as observed in this study is outlined in Fig 5.

## Supporting information

S1 Fig Gel analysis picture showing genotyping of miR-9 transgenic mice. Mice tail DNA was extracted and amplified using specific primers to detect incorporated miR-9. (Forward primer --5’-GCCCTGCTGATACCAAGTG-3’; reverse primer 5’-GTGCGGCTAGAACATCCA-3’). Agarose gel analysis confirmed presence of 377bp band in the transgenic animals.
(PPTX)

S2 Fig Confirmation of cellular phenotypes following isolation from C57BL/6 mice heart. RT-PCR showing A) very high levels of CD31 mRNA expression in the isolated ECs, which were subsequently used. Similarly, B) FSP1 showed high level of expression in the fibroblasts and C) β-MHC was highly expressed in the myocytes [MH = mouse heart; EC = endothelial cell; Fib = fibroblasts; Myo = myocyte, β-MHC = myosin heavy chain; FSP = fibroblast-specific protein 1, PCR primers for this assay have been listed in S1 Table].
(PPTX)

S1 Table The primers for the experiments. (DOCX)
