# Supplementary figures and images for: Endothelial derived miRNA-9 mediated cardiac fibrosis in diabetes and its regulation by ZFAS1
Source: PLoS One. 2022 Oct 14;17(10):e0276076. doi: 10.1371/journal.pone.0276076 (PMC9565427; doi:10.1371/journal.pone.0276076)

## Slide 1
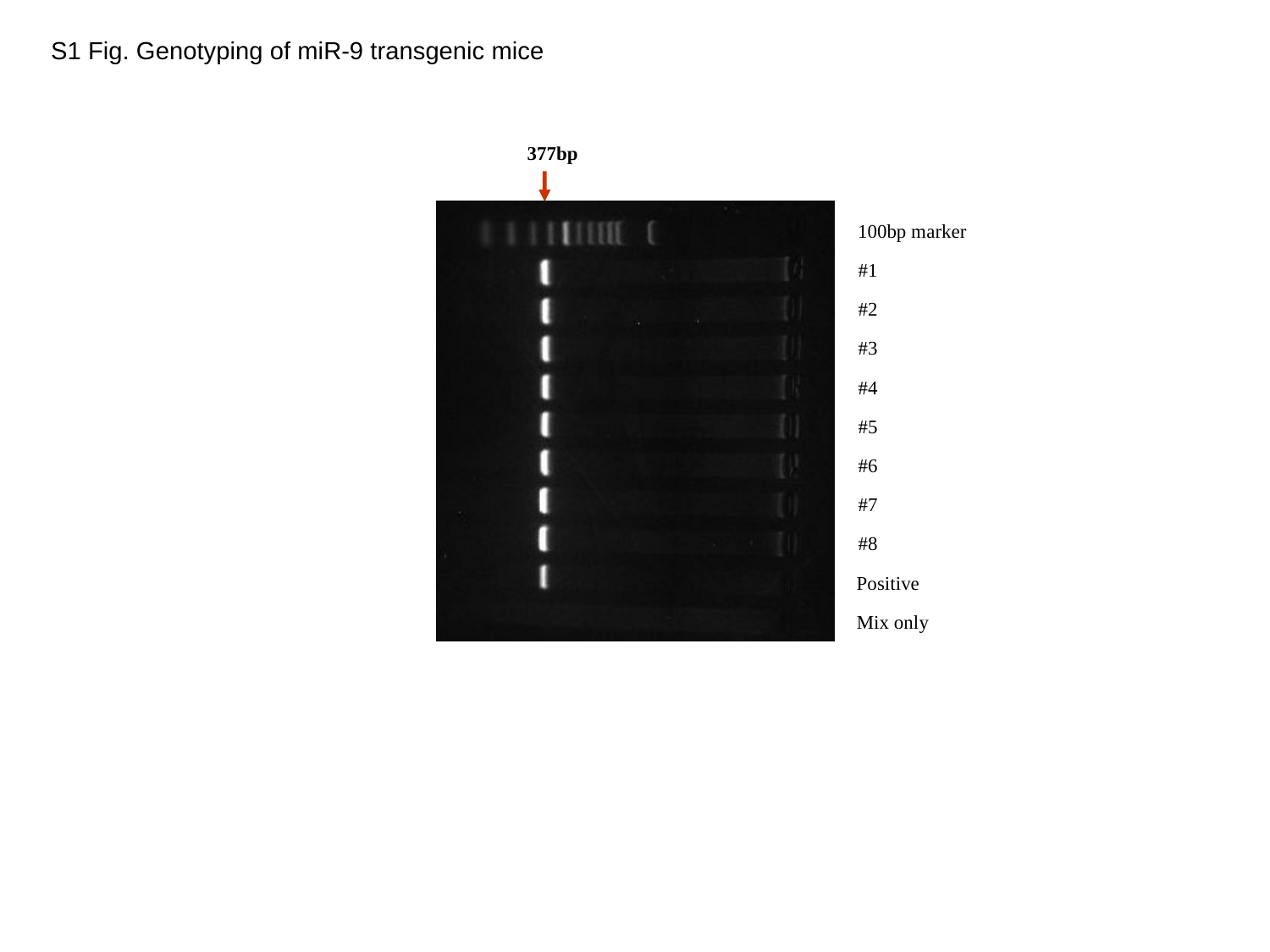

S1 Fig. Genotyping of miR-9 transgenic mice
377bp
100bp marker
#1
#2
#3
#4
#5
#6
#7
#8
Positive
Mix only

Supplement: S1 Fig — Mice tail DNA was extracted and amplified using specific primers to detect incorporated miR-9. (Forward primer --5’-GCCCTGCTGATACCAAGTG-3’; reverse primer 5’-GTGCGGCTAGAACATCCA-3’). Agarose gel analysis confirmed presence of 377bp band in the transgenic animals. (PPTX) [file pone.0276076.s001.pptx]
