# Supplementary material for: Endothelial derived miRNA-9 mediated cardiac fibrosis in diabetes and its regulation by ZFAS1
Source: PLoS One. 2022 Oct 14;17(10):e0276076. doi: 10.1371/journal.pone.0276076 (PMC9565427; doi:10.1371/journal.pone.0276076)
Supplement: S2 Fig — RT-PCR showing A) very high levels of CD31 mRNA expression in the isolated ECs, which were subsequently used. Similarly, B) FSP1 showed high level of expression in the fibroblasts and C) β-MHC was highly expressed in the myocytes [MH = mouse heart; EC = endothelial cell; Fib = fibroblasts; Myo = myocyte, β-MHC = myosin heavy chain; FSP = fibroblast-specific protein 1, PCR primers for this assay have been listed in S1 Table]. (PPTX) [file pone.0276076.s002.pptx]

## Slide 1
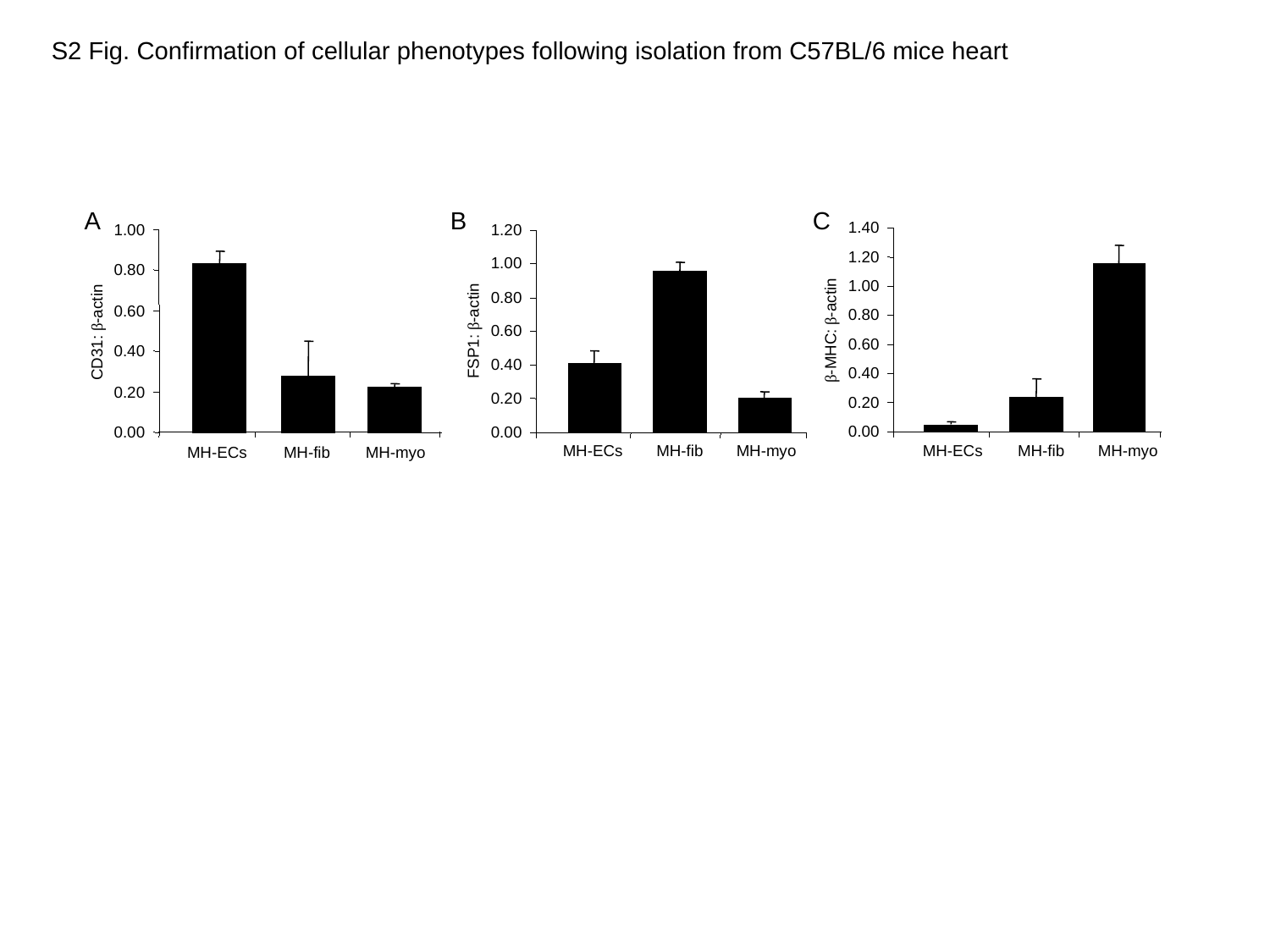

S2 Fig. Confirmation of cellular phenotypes following isolation from C57BL/6 mice heart
A
B
C
1.40
1.20
1.00
0.80
β-MHC: β-actin
0.60
0.40
0.20
0.00
MH-ECs
MH-fib
MH-myo
1.00
0.80
0.60
CD31: β-actin
0.40
0.20
0.00
MH-ECs
MH-fib
MH-myo
1.20
1.00
0.80
0.60
FSP1: β-actin
0.40
0.20
0.00
MH-ECs
MH-fib
MH-myo
