# Supplementary material for: Endothelial derived miRNA-9 mediated cardiac fibrosis in diabetes and its regulation by ZFAS1
Source: PLoS One. 2022 Oct 14;17(10):e0276076. doi: 10.1371/journal.pone.0276076 (PMC9565427; doi:10.1371/journal.pone.0276076)
Supplement: S1 Table — (DOCX) [file pone.0276076.s003.docx]

S1 Table: The primers for the experiments

___________________________________________________________________________

Gene Primer sequence (5’-3’) Product size

__________________________________________________________________________

Mmu CD31 ACCGGGTGCTGTTCTATAAGG 194bp

CACCTTGGGCTTGGATACGC

Mmu FSP1 GTCCACCTTCCACAAATACTC 147bp

AAGTTGCTCATCACCTTCTGG

Mmu β-MHC CCTCCTCACATCTTCTCCATCTCT 94bp

CTCCGGATTCTCCGGTGAT

__________________________________________________________________________

Note: Mmu= mouse; β-MHC, myosin heavy chain
